# Optimizing maternal and neonatal outcomes with postpartum contraception: impact on breastfeeding and birth spacing

**DOI:** 10.1186/s40748-016-0040-y

**Published:** 2017-01-13

**Authors:** Aparna Sridhar, Jennifer Salcedo

**Affiliations:** 1 Department of Obstetrics and Gynecology, David Geffen School of Medicine at University of California Los Angeles, California, USA; 2 Department of Obstetrics, Gynecology & Women’s Health, University of Hawaii John A. Burns School of Medicine, Hawaii, USA

**Keywords:** Breastfeeding, Contraception, Inter-pregnancy interval, Lactation, Postpartum

## Abstract

Postpartum contraception is important to prevent unintended pregnancies. Assisting women in achieving recommended inter-pregnancy intervals is a significant maternal-child health concern. Short inter-pregnancy intervals are associated with negative perinatal, neonatal, infant, and maternal health outcomes. More than 30% of women experience inter-pregnancy intervals of less than 18 months in the United States. Provision of any contraceptive method after giving birth is associated with improved inter-pregnancy intervals. However, concerns about the impact of hormonal contraceptives on breastfeeding and infant health have limited recommendations for such methods and have led to discrepant recommendations by organizations such as the World Health Organization and the U.S. Centers for Disease Control and Prevention. In this review, we discuss current recommendations for the use of hormonal contraception in the postpartum period. We also discuss details of the lactational amenorrhea method and effects of hormonal contraception on breastfeeding. Given the paucity of high quality evidence on the impact on hormonal contraception on breastfeeding outcomes, and the strong evidence for improved health outcomes with achievement of recommended birth spacing intervals, the real risk of unintended pregnancy and its consequences must not be neglected for fear of theoretical neonatal risks. Women should establish desired hormonal contraception before the risk of pregnancy resumes. With optimization of postpartum contraception provision, we will step closer toward a healthcare system with fewer unintended pregnancies and improved birth outcomes.

## Background

Postpartum contraception is important to prevent unintended pregnancies and short intervals between pregnancies. The appropriate method and timing of contraception initiation following a birth, miscarriage or pregnancy termination depends on multiple factors such as a patient’s personal preferences, medical history, risk for pregnancy, breastfeeding preferences, and access to contraceptive services. The most important role of postpartum contraception is to help a woman achieve the desired interval before the next pregnancy in order to optimize her health and that of her young children. Theoretical concerns regarding the impact of contraception on breastfeeding must be appropriately weighed against well-supported impacts on inter-pregnancy intervals and the woman’s informed decisions regarding her reproductive health.

### Inter-pregnancy interval and perinatal outcomes

Assisting women in achieving recommended inter-pregnancy intervals (IPIs), defined as the time interval between a live birth and the beginning of the next pregnancy, is a significant maternal-child health concern. Short IPIs are associated with negative perinatal, neonatal, infant, and maternal health outcomes. The concept of an ideal inter-pregnancy interval emerged from a report published by World Health Organization (WHO) in 2005. Based on the best available evidence at that time, the experts reached a consensus of 24 months as the IPI. This interval was consistent with the joint WHO and United Nations Children’s Emergency Fund (UNICEF) recommendation that women breastfeed for at least 2 years [1].

Recommendations from the WHO report, “Effect of Interpregnancy Interval on Adverse Perinatal Outcomes,” were evaluated using the Perinatal Information System Database of the Latin American Center for Perinatology and Human Development from 1985 to 2004. Compared to infants with IPIs of 18–23 months, those born to women with intervals shorter than 6 months had an increased risk of many adverse neonatal and perinatal outcomes [2]. A systematic review by Conde-Agudelo et al. that included 77 studies conducted in countries across six continents analyzed the association of IPIs with outcomes such as preterm birth, low birth weight, small size for gestational age (SGA) at birth, fetal death, and early neonatal death. For IPIs shorter than 6 months, there were significantly increased risks for preterm birth (Odds Ratio = 1.40), SGA (Odds Ratio =1.26), and low birth weight (Odds Ratio = 1.61). Intervals of 6 to 17 months were also associated with a significantly greater risk for these three adverse perinatal outcomes. Additionally, among women with previous low-transverse caesarean sections who underwent trials of labor, there was noted to be increased risk of uterine rupture with IPIs less than 16 months [3]. Another recent study analyzing all live births between 1991 and 2010 in California concluded that women with IPIs of less than 1 year following live birth were at increased risk for preterm birth [4]. No conclusions on the impact of IPI on maternal mortality and morbidity were able to be drawn in the WHO’s report due to limited available data [1].

Unfortunately, data from the 2015 National Vital Statistics Report and the National Survey of Family Growth demonstrate that in the United States 30% of women experience IPIs of less than 18 months. Short IPIs in the U.S. are inversely associated with maternal age, with more than two thirds (67%) of teenagers between ages 15–19 experiencing IPIs of less than 18 months [5].

### Postpartum endocrine changes

To understand the timing and mechanisms by which women resume risk of pregnancy following delivery, it is important to review the cascade of endocrine changes that take place after parturition. Immediately following delivery, the inhibitory effect of the estrogen and progesterone levels of pregnancy decreases and the pulsatile activity of the pituitary follicular stimulating hormone (FSH) and luteinizing hormone (LH) resumes [6]. In non-lactating women, studies evaluating urinary pregnanediol levels have reported a mean first ovulation ranging from 45 to 94 days postpartum, with the earliest ovulation reported 25 days after delivery [7, 8]. Consequently, women are assumed to be protected from pregnancy for 4 weeks following delivery. Similarly, studies looking at ovulation after abortion suggest that most women ovulate before resuming menses, with mean time to ovulation of 22 days [9]. Lactation extends the period of postpartum infertility. Nerve impulses arising from the nipple and areola due to infant suckling release prolactin from the hypothalamus. This, in turn, suppresses the pulsatile release of gonadotropin-releasing hormone (GnRH) by the hypothalamus, likely by increasing beta-endorphin production. The pulsatile secretion of GnRH is necessary to stimulate the cells in anterior pituitary to produce FSH and LH needed for ovulation. Thus continued suckling and lactation provide protection from pregnancy [10].

### Lactational amenorrhea method

The lactational amenorrhea method (LAM) is the specific name given to use of breastfeeding as a dedicated method of contraception. For breastfeeding to serve as an effective method of contraception, the woman must be exclusively or nearly exclusively breastfeeding (at least 85% of infant feeding coming from breastfeeding), be within the first 6 months following delivery, and remain amenorrheic. Some experts additionally believe that milk expression by hand or pump does not retain the same fertility-inhibiting effect as infant nursing [11]. Clinical studies of the contraceptive effect of LAM have demonstrated cumulative 6-month life-table perfect-use pregnancy rates of 0.5 – 1.5% among women who relied solely on LAM. A Cochrane review published in 2015 estimated the typical use failure rate of LAM to be 0.45 – 7.5% [12–15].

Although LAM is a highly effective temporary method of contraception, rates of the exclusive breastfeeding required for its effectiveness are low in the United States. Data from the National Immunization Survey describes an 80% incidence of breastfeeding initiation, declining to a 4 weeks postpartum exclusive breastfeeding rate of 54%, which declines to 20% at 6 months [16]. According to this data, exclusive breastfeeding rates at 3 months were lower in non-white, unmarried women with lower socio-economic status compared to non-Hispanic white, well educated, married women [16]. Among the individual states, Montana had the highest (60%) and Mississippi the lowest (21%) exclusive breastfeeding rates at 3 months in 2013 [17].

Historically, women have also been assumed to experience protection from pregnancy during the traditionally recommended 6-week period of pelvic rest following delivery. While 6-weeks may have been historically recommended to accommodate the expected time period of uterine involution, and due to its concurrent timing with the historically recommended 6-week postpartum visit, no evidence supports any specific interval of post-delivery abstinence. McDonald et al. [18] analyzed a prospective cohort of approximately 1500 nulliparous women to investigate the timing of resumption of vaginal sex after childbirth and noted that 41% of women admitted to resuming intercourse by 6 weeks, while 65 and 78% endorsed vaginal sex by 8 and 12 weeks postpartum, respectively. Spontaneous vaginal birth with an episiotomy or laceration, forceps- or vacuum-assisted vaginal birth, cesarean section, and breastfeeding are negatively associated with resumption of intercourse following delivery, while young age (<25 years) and living with a partner are associated with earlier resumption of intercourse [18].

### Contraception provision postpartum

**Fig. 1:**
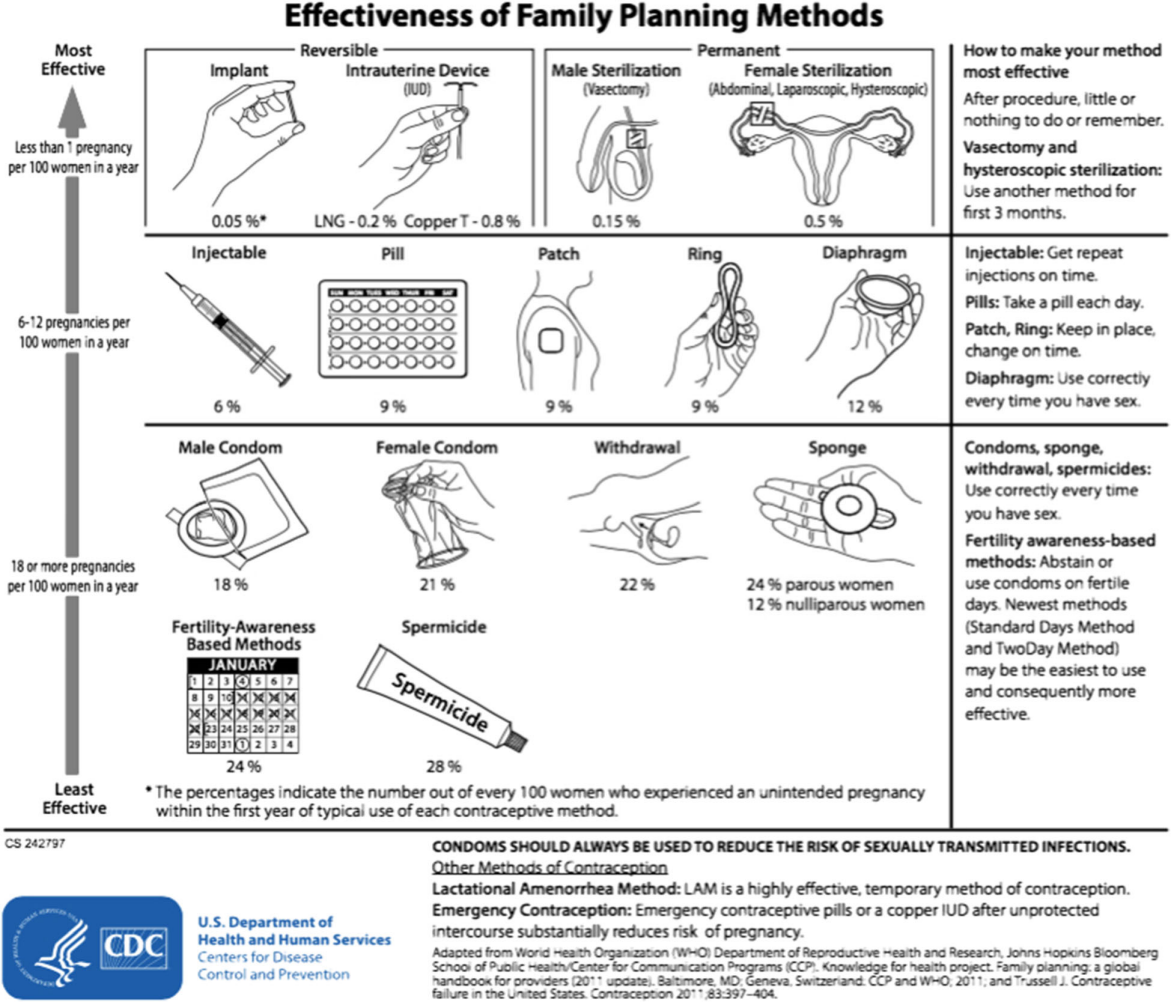
Effectiveness of Family Planning Methods (Adapted from Centers for Disease Control and Prevention)

Given that women resume risk for pregnancy from 4 weeks to 6 months postpartum, effective contraceptive methods must be available to assist women in reaching recommended IPIs. Figure 1 demonstrates currently available contraceptive methods in the U.S. and their associated effectiveness in typical use. Provision of any contraceptive method within 90 days of giving birth is associated with improved IPIs [19]. However, use of long-acting reversible contraception methods (LARC) such as intrauterine devices (IUDs) and the subcutaneous contraceptive implant, both with effectiveness that is generally not reduced by user error, have been shown to substantially improve IPIs compared to other methods. More specifically, women using LARC methods after delivery have 3.89 times the likelihood of reaching recommended birth spacing intervals compared to women using condoms only, while women using user-dependent hormonal methods (pill, patch, vaginal ring and injection) have 1.89 times the likelihood of achieving recommended spacing compared to barrier method users [19, 20].

### Recommendations by the World Health Organization and Centers for Disease Control and Prevention

Despite evidence that early initiation of postpartum contraception increases IPIs, concerns about the impact of hormonal contraceptives on breastfeeding and infant health have limited recommendations for such methods and have lead to discrepant recommendations by organizations such as the World Health Organization (WHO) and the U.S. Centers for Disease Control and Prevention (CDC). While both the WHO and CDC generally agree that the initiation of estrogen-containing methods should be delayed for 3–6 weeks postpartum (depending on a woman’s medical risk factors) until the risk of venous thromboembolism (VTE) decreases to approximately the non-pregnant baseline, the WHO has issued more conservative recommendations than the CDC regarding use of both estrogen-containing and progestin-only methods by breastfeeding women [21, 22].

In the WHO Medical Eligibility Criteria for Contraceptive Use (MEC), Fifth Edition [23] estrogen-containing contraceptives (including combined oral contraceptives, the patch, and the vaginal ring) are considered to pose unacceptable health risks (Category 4) when used by breastfeeding women within the first 6 postpartum weeks. These methods are considered by the WHO to have theoretical or proven risks that usually outweigh their advantages (Category 3) until breastfeeding women are at least 6 months postpartum. In contrast, in the U.S. Centers for Disease Control and Prevention Medical Eligibility Criteria for Contraceptive Use (CDC MEC), the advantages of using such estrogen-containing methods are stated to generally outweigh the theoretical or proven risks (Category 2) for women without complicating medical conditions starting 6 weeks after delivery [21] (Table 1).

**Table 1:** Medical Eligibility Criteria [CDC/WHO]

Method	<10 min	<48 h	<21 days	21 to <30 days	30-42 days	42 days-6 months	>6 months
Breastfeeding Women	Category [CDC/WHO]						
Combined hormonal contraceptives	4/4	4/4	4/4	3/4	2^a^/4	2/3^c^	2/2
Progestin-only pills	2/2	2/2	2/2	2/2	1/2	1/1	1/1
DMPA	2/3	2/3	2/3	2/3	1/3	1/1	1/1
Etonogestrel implant	2/2	2/2	2/2	2/2	1/2	1/1	1/1
Levonorgestrel intrauterine device	2/2	2/2	2/3	2^b^/3^b^	1^b^/1^b^	1/1	1/1
Copper intrauterine device	1/1	2/1	2/3	2^b^/3^b^	1^b^/1^b^	1/1	1/1
Nonbreastfeeding Women	Category [CDC/WHO]						
Combined hormonal contraceptives	4/3^d^	4/3^d^	4/3^d^	2^a^/2^a^	2^a^/2^a^	1/1	1/1
Progestin-only pills	1/1	1/1	1/1	1/1	1/1	1/1	1/1
DMPA	1/1	1/1	1/1	1/1	1/1	1/1	1/1
Etonogestrel implant	1/1	1/1	1/1	1/1	1/1	1/1	1/1
Levonorgestrel intrauterine device	1/1	2/1	2/3	2^b^/3^b^	1^b^/1^b^	1/1	1/1
Copper intrauterine device	1/1	2/1	2/3	2^b^/3^b^	1^b^/1^b^	1/1	1/1

^a^ CDC & WHO Category 3 for women with other risk factors for VTE: 35 years old or older, previous VTE, thrombophilia, immobility, peripartum transfusion, peripartum cardiomyopathy, obesity, peripartum hemorrhage, cesarean delivery, preeclampsia, or smoking

^b^ Refers to 28 days for intrauterine device insertion timing

^c^ Refers to women who are primarily breastfeeding

^d^ WHO Category 4 for women with other risk factors for VTE

In the following sections we provide a detailed review of the impact of each hormonal contraceptive method on breastfeeding outcomes.

#### a) Combined oral contraceptives

A CDC review of combined oral contraceptives from 2015 included 15 articles from 13 studies evaluating the impact of estrogen-containing oral contraceptive pills on breastfeeding and associated infant outcomes. Unfortunately, applicable studies were noted to be of only poor to fair methodological quality and many were from earlier decades when the estrogen-content of combined oral contraceptives was substantially higher than in modern pills [24]. In this review, no studies found significant impact on infant weight gain when combined oral contraceptives (COCs) were initiated at 6 weeks or later postpartum, and none found negative impact on other infant health outcomes regardless of the timing of COC initiation [24]. However, the results of studies examining the impact of COCs on breastfeeding performance were inconsistent [24]. A study by the WHO in 1984 assigned women to a COC containing 30 micrograms of ethinyl estradiol and 150 micrograms of levonorgestrel or a progestin-only pill containing 75 micrograms of norgestrel, started after 6 weeks postpartum. Breast milk volume was quantified following pump expression. Mean breast milk volume was lower in the COC group at 9, 16 and 24 weeks by 18–25 mL. However, no differences were noted between groups on use of supplemental infant nutrition [25]. In 2012, Espey et al. performed a randomized controlled trial of breastfeeding women following term delivery who desired to initiate oral contraception. At two weeks postpartum women initiated either a combined pill of 35 mcg ethinyl estradiol and 1 mg norethindrone (*n* = 64) or a progestin-only pill (POP) containing 35 micrograms of norethindrone (*n* = 63) [26]. There was no significant difference between the COC and POP groups in the primary outcome of breastfeeding continuation over the 6 months of follow-up, nor were there differences found in infant growth parameters, satisfaction with breastfeeding or oral contraceptive use, perception of milk supply adequacy, formula supplementation, or reasons cited for discontinuing breastfeeding or oral contraceptive pills [26]. Given that in non-breastfeeding women without contraindications to estrogen, combined oral contraceptives have been found to have better efficacy, higher continuation rates, and fewer side effects than POPs, and that 35 micrograms is the highest estrogen content in commonly used COCs, the authors conclude that it is reassuring that combined pills do not have a major impact on breastfeeding continuation or infant growth and a larger equivalency study should be performed to clarify the clinical impact of COCs on lactation [26]. No randomized controlled trials are available that evaluate the other estrogen-containing contraceptives available in the U.S., the contraceptive patch and vaginal ring, which have different hormonal content and absorption profiles than combined oral contraceptive pills [25].

#### b) Progestin only contraceptives

In contrast to estrogen-containing contraceptives, the WHO considers the benefits of progestin-only oral contraceptives and the progestin-containing contraceptive implant to generally outweigh risks of these methods during the first 6 postpartum weeks for breastfeeding women (Category 2), but recommends that the contraceptive injection (depot medroxyprogesterone acetate [DMPA] in the U.S.) be initiated no sooner than 6 weeks postpartum [23]. In contrast, the CDC considers the benefits of all progestin-only systemic methods to outweigh potential risks during the first 30 days postpartum for breastfeeding women (Category 2), and states that all such methods may be used without restriction (Category 1) 30 days following delivery [21].

While the WHO acknowledges that available evidence does not support a negative effect of progestin-only contraceptives on breastfeeding performance, and has generally demonstrated no negative impacts on infant growth or development, concern over lack of evidence on potential longer-term pediatric impacts have led to conservative recommendations for hormonal contraceptive use, particularly for contraceptive injections during the early postpartum period [23]. Animal data has suggested some effect of progesterone on the developing brain, but whether such effects are present in humans, and of what potential consequences, are unclear [23, 27]. Although limited comparative studies have not noted a difference in breastfeeding or infant outcomes with exposure to progestin-injections compared to other progestin-only methods, the higher maternal systemic hormone levels attained with progestin-injections (compared to other systemic progestin-only methods) have led the WHO Guideline Development Group to be concerned about infant hormone exposure during use of these methods, particularly in the first 6 weeks [23]. Additionally, given the paucity of high quality evidence in this area, WHO and CDC expert opinion may understandably differ based on issues such as the difference in exclusive breastfeeding rates between U.S. women and those in less developed parts of the world and the availability of safe alternatives to breast milk for infant and early childhood nutrition.

While the steroids progesterone and nestorone are orally inactive, progestin-containing contraceptives currently used in the United States contain orally active progestins [28]. The amount of progestin transferred to an infant through breast milk by hormonal contraceptive users is variable. For contraceptive implant users, the mean concentration of etonogestrel in breast milk ranges from 405 to 548 pmol/L, approximately one-half the maternal plasma concentration, which results in transferring of approximately 75–120 nanograms of hormone per day to the fully breastfed infant [28, 29]. This daily progestin dose is similar to the range estimated for progestin-only pills, which is approximately twice that of levonorgestrel-releasing IUDs. It is significantly lower than that received by infants whose mothers use progestin injectables, which is in the range of 1–13 micrograms, approximately 88% of the serum concentration [28, 30–32].

The issue of infant exposure to exogenous hormones is further complicated by the fact that newborns are initially unable to either absorb or metabolize exogenous hormones, followed by an ability to metabolize more effectively than absorb, then an ability to do both successfully by about 24 weeks of gestation [33]. For women using levonorgestrel-releasing IUDs, compared to non-hormonal IUDs, no differences were noted in infants’ serum electrolytes, protein, creatinine, iron, cholesterol, or liver enzyme levels [31]. Similarly, no differences in the biochemical markers of FSH, LH, testosterone, or immunoglobin differences were noted between male infants of progestin-only pill users or users of a progestin-implant compared to non-hormonal contraceptive users [34, 35].

An updated review on progestin-only contraceptives and breastfeeding by Phillips et al. in 2015 noted that 11 non-randomized trials and observational studies found either no effect or a positive effect on breastfeeding outcomes with use of progestin-injectables initiated within the first 6 weeks postpartum [36] Similarly, eight observational studies on progestin-only pills and breastfeeding found either no differences or improved breastfeeding outcomes when pills were initiated within 6 weeks postpartum [36]. A randomized controlled trial noted no difference in infant weight gain at 14 days compared to infants not exposed to hormonal contraception [37, 38]. Similarly, in studies examining infant growth effects when progestin-only methods are initiated after 6 weeks postpartum, most have noted no differences, although small differences in both directions have been found [39].

In a study by Reinprayoon et al., women who initiated an etonogestrel implant while fully breastfeeding were found to have similar breast milk volume, composition, rates of initiation and timing of supplemental feedings, and infant growth characteristics as women who initiated a non-hormonal IUD [29]. Similarly, for women using a levonorgestrel-releasing IUD, compared to women using non-hormonal IUDs, mean duration of breastfeeding was found to be similar, although slightly fewer women were still breastfeeding at 75 days in the levonorgestrel group. No differences were noted in the growth, health, or developmental milestones measured [32].

In addition to infant hormone exposure during the first 6 weeks postpartum, some experts have expressed concern that immediate postpartum initiation of hormonal contraceptives prior to the natural postpartum decline of progesterone may have greater impact on breastfeeding performance than methods initiated even in the early weeks postpartum. Most recently, a randomized trial by Chen et al. comparing postplacental to delayed levonorgestrel IUD insertion at 6–8 weeks noted that women in the delayed group were more likely to still be breastfeeding at 6 months postpartum, although no differences in breastfeeding initiation or continuation at 6–8 weeks or at 3 months was seen [40]. This difference persisted when women who never breastfed were excluded and when only primiparous women without previous breastfeeding experience were considered [40]. A biologic etiology for this difference only seen at 6 months is difficult to ascertain given the low systemic levels of levonorgestrel found in levonorgestrel-IUD users and the fact that any impact of exogenous hormones on breastfeeding would seem most plausible at or surrounding breastfeeding initiation.

Such a negative impact of immediate hormonal LARC placement on breastfeeding outcomes has not been found in studies evaluating the etonogestrel contraceptive implant [41, 42]. For instance, a study comparing initiation of the contraceptive implant within 3 days postpartum, compared to 4–8 weeks postpartum, found no significant difference between full or any breastfeeding at any time point, breast milk creamatocrit, or a clinically significant difference in time to lactogenesis [42]. Another study that used deuterium (an isotope of hydrogen that is used as a tracer) to compare milk intake between infants whose mothers had a Nexplanon placed within 48 h of delivery to those using no contraceptive method found no difference in milk intake at any time within 6 weeks and no difference in newborn weight between groups during the follow-up period [43].

Data on the impact of DMPA administration before postpartum discharge is mixed but generally reassuring. While a study of Peruvian women noted that women who initiated DMPA more than 72 h postpartum were more likely than both women who initiated DMPA within 72 h postpartum and those who didn’t initiate DMPA postpartum to be exclusively breastfeeding at 3 months, DMPA use overall was associated with higher exclusive breastfeeding rates at 3 months compared with non-use [44]. The authors of this study postulated that increased contact with healthcare providers by women who initiated DMPA more than 72 h following delivery may have provided additional opportunities for breastfeeding support [44]. In contrast, a prospective non-randomized trial comparing progestin-only contraceptive administration before postpartum discharge with non-hormonal methods found that women who initiated DMPA before postpartum discharge saw no difference in breastfeeding continuation rates at 2 weeks or 6 weeks, and no difference in discontinuation due to perceived inadequate milk supply, despite the fact that women in the DMPA group were less likely to breastfeed based on their younger age and lower gravidity and parity [45]. Study authors concluded that women who choose hormonal methods are likely to be different in breastfeeding likelihood than women who initiate non-hormonal methods or no method [45]. Similarly, a retrospective cohort study by Brownell et al. found no significant difference in breastfeeding cessation within 2 or 6 weeks postpartum between women who initiated DMPA before postpartum discharge (a mean of 37 h following delivery) and women who did not initiate DMPA, but did note that the DMPA group was significantly less likely to have a timed breastfeeding goal (such as planning to breastfeed until a particular infant age or developmental milestone) and to report social support for breastfeeding. These findings support the idea that women who choose to initiate hormonal contraception before postpartum discharge may be different in important ways from women who make other postpartum contraceptive choices [46]. The American College of Obstetricians & Gynecologists (ACOG) recognizes that clinical judgment must weigh the need for contraception against the theoretical neonatal risks and endorses early initiation of DMPA postpartum in indicated clinical situations, such as a high risk of being lost to postpartum follow-up [47].

#### c) Emergency contraceptive pills

In addition to routine contraception, emergency contraception is a promising component of a comprehensive contraceptive strategy to reach recommended birth spacing intervals. Emergency contraceptive pills (ECPs) are pills taken as soon as possible, and within 120 h, after unprotected or inadequately protected intercourse in order to reduce the risk of pregnancy when a routine contraceptive method was not used or was not used correctly. Two types of dedicated ECP products are available in the U.S. They are progestin-only ECPs comprised of 1.5 mg levonorgestrel, and 30 mg of ulipristal acetate, which is a progestin-receptor modulator. Both medications work by delaying or preventing ovulation and neither has evidence for post-fertilization effects [48]. Ulipristal acetate has been found to be more efficacious overall, and specifically for overweight and obese women and women who require ECPs for intercourse occurring close to ovulation [49, 50]. In both the WHO and CDC MEC, levonorgestrel ECPs are classified as Category 1 for all users [21, 23]. However, the WHO MEC classifies use of ulipristal acetate by breastfeeding women as Category 2 and recommends that women avoid breastfeeding for 1 week following use, discarding breast milk during that time, based on the package labeling for UPA which states that, “Use of ella® by breastfeeding women is not recommended,” since it is “unknown if ulipristal acetate is excreted in human milk [23, 51].” In contrast, the CDC MEC classifies ulipristal acetate use by breastfeeding women as Category 1, but recommends that breast milk be expressed and discarded for 24 h following use. Since there are no studies evaluating the impact of ulipristal acetate exposure on infants [52], the recommendation to discard breast milk for 24 h is based on rapidly declining levels of ulipristal acetate and its metabolite measured in breast milk during this time period.

Professional organizations such as the ACOG and the American Academy of Pediatrics (AAP) recommend advance prescription of emergency contraceptive pills to all reproductive aged women [53, 54]. In a randomized trial of breastfeeding Egyptian women who planned to use LAM and delay a next conception by at least 1 year, women in the standard LAM counseling group were significantly more likely than women in the levonorgestrel ECP advanced provision group to experience pregnancy within 6 months postpartum (of which 80% were unplanned and/or undesired), despite similar rates of unprotected intercourse after expiration of LAM criteria in both groups [55]. Unfortunately, knowledge of emergency contraception by postpartum women is low. In a study of inner city postpartum women, only 36% had heard of emergency contraception, only 19% could name or describe a method of emergency contraception, and only 7% could identify correct timing of ECP use [56]. Of those who were aware of emergency contraception, only 44% thought it was safe, despite CDC MEC Category 1–2 classification, and 32% incorrectly believed that it served as an abortifacient [56]. However, two-thirds of women in the study stated that they would be willing to use emergency contraception in the future [56].

### System based barriers for postpartum contraception use

Despite the effectiveness of currently available contraception, U.S. women seeking to establish postpartum contraception currently face a myriad of systems barriers. Of women who indicate a plan for postpartum IUD placement, only 23–60% undergo insertion as planned [57, 58]. Women are often denied inpatient LARC placement due to the fact that reimbursement for all delivery-related care is generally based on a global fee that does not carve out the cost of LARC devices or insertions [59, 60]. This reimbursement scheme severely disincentivizes hospitals to supply and dispense LARC devices, which typically have wholesale costs upwards of $600 [59]. While Medicaid and most private insurance plans have been able to separate out reimbursement for sterilization during a delivery-related hospitalization, currently only a small minority of states have been able to address the issue for LARC devices, and primarily only for Medicaid-covered patients [59, 60]. Interestingly, this slow systems-based change takes place in a setting in which substantial cost-savings to public programs facilitating inpatient LARC placement has been demonstrated [60]. Further barriers are encountered by women who receive delivery-related care in Catholic hospitals, which currently represent one-sixth of all hospital beds in the U.S., as these facilities do not permit placement of LARC devices for the purpose of contraception [59].

Additionally, postpartum women often receive inconsistent, and sometimes incorrect, information from various members of the healthcare team regarding the safety and impact of contraception on breastfeeding. For instance, in a recent survey by Dunn et al., while 77% of lactation consultants reported offering advice about postpartum contraception and its impact on breastfeeding, the vast majority stated that progestin-only methods used in the first 21 days following delivery had theoretical or proven risks that outweighed benefits or presented unacceptable health risks (equivalent to MEC Category 3 or Category 4), which is inconsistent with medical professional guidelines and the best available evidence [61]. Specifically, 76.3% stated that progestin-only pills ought to be avoided during this time period, while 90.1% stated the same for DMPA and 92.1% for the etonogestrel implant [61]. The inconsistencies found in this study are particularly concerning, given that lactation consultants are generally considered the premier experts on breastfeeding on postpartum inpatient units where many women are finalizing contraceptive choices for initiation before discharge.

## Conclusion

Regardless of age and intended family size, unintended pregnancy can occur at any point in a woman’s reproductive life. As women spend the majority of their reproductive years attempting to avoid unintended pregnancy, contraception counseling is an important aspect women’s healthcare, especially for those who have recently experienced pregnancy. There are very few non-hormonal contraceptive options available in the United States, and most are associated with high failure rates in typical use. Healthcare providers must communicate with women about the typical use failure rates of contraceptives and assist patients in initiating the most effective method that best fits their medical situation and preferences. Providers must also be familiar with USMEC and US Selected Practice Recommendations for Contraceptive Use in order to initiate and manage post-pregnancy contraception safely and appropriately [21]. Further, many women use contraceptive methods for their non-contraceptive benefits and may need to initiate or resume methods for such purposes postpartum. The USMEC clarifies that recommendations refer to the safety of contraceptive methods when used for contraceptive purposes and do not apply to the use of contraceptive methods for treatment of medical conditions (as the eligibility criteria may differ in such circumstances) [21].

Women may wish to discuss contraception both prenatally and after hospital discharge. A multicenter  randomized controlled study evaluating antenatal discussion of planned postpartum contraception failed to demonstrate significant effect on contraceptive use or subsequent pregnancy rates [62]. An evaluation of postpartum contraception educational method by the Cochrane database also failed to identify specific strategies that work universally to improve awareness of women in this regard. Thus, to date, research has not reached a consensus on the optimal timing and method to discuss postpartum contraception with patients [63].

Despite the paucity of high quality evidence of the impact on hormonal contraception on breastfeeding outcomes, and the strong evidence for improved health outcomes with achievement of recommended birth spacing intervals, some authors have gone as far as to suggest that conducting a controlled trial to evaluate the effects of timing of hormonal contraception initiation on lactation would be unethical given the primacy of non-hormonal methods in breastfeeding and the fact that hormonal contraceptives could theoretically have negative impacts [64]. However, other authors have advocated strongly that the real risk of unintended pregnancy and its consequences must not be neglected for fear of the theoretical neonatal risks, and that women should establish desired hormonal contraception before the risk of pregnancy resumes, which is generally 4 weeks following delivery unless criteria for the lactational amenorrhea method is met [65].

Women who desire a contraceptive method should have it available as soon as possible. Advanced provision of EC is recommended by professional societies and may serve an important role postpartum. Systems barriers to women’s ability to achieve recommended birth spacing must be addressed. With optimization of postpartum contraception provision, we will step closer toward a healthcare system with fewer unintended pregnancies and improved birth outcomes.

## Abbreviations

CDC MEC: Centers for Disease Control and Prevention Medical Eligibility Criteria for Contraceptive Use
CDC: Centers for Disease Control and Prevention
COC: Combined oral contraceptives
DMPA: Depot medroxyprogesterone acetate
ECP: Emergency contraceptive pills
FSH: Follicular stimulating hormone
GnRH: Gonadotropin-releasing hormone
IPI: Inter-pregnancy interval
IUD: Intrauterine device
LAM: Lactational amenorrhea method
LARC: Long-acting reversible contraception
LH: Luteinizing hormone
POP: Progestin-only pill
SGA: Small size for gestational age
UNICEF: United Nations Children's Emergency Fund
VTE: Venous thromboembolism
WHO MEC: World Health Organization Medical Eligibility Criteria for Contraceptive Use
WHO: World Health Organization
